# Acute Liver Toxicity due to Efavirenz/Emtricitabine/Tenofovir

**DOI:** 10.1155/2015/280353

**Published:** 2015-06-16

**Authors:** Rashmee Patil, Mel A. Ona, Haris Papafragkakis, Jeanne Carey, Yitzchak Moshenyat, Adib Alhaddad, Sury Anand

**Affiliations:** ^1^Department of Internal Medicine, NYU Lutheran Medical Center, Brooklyn, NY 11220, USA; ^2^Department of Gastroenterology & Hepatology, The Brooklyn Hospital Center, Brooklyn, NY 11201, USA; ^3^Department of Infectious Diseases, NYU Lutheran Medical Center, Brooklyn, NY 11220, USA; ^4^Department of Gastroenterology & Hepatology, NYU Lutheran Medical Center, Brooklyn, NY 11220, USA

## Abstract

The fixed-dose combination of Efavirenz/Emtricitabine/Tenofovir is a first-line agent for the treatment of HIV; however few cases have reported hepatotoxicity associated with the drug. We report a case of Efavirenz/Emtricitabine/Tenofovir-associated hepatotoxicity presenting mainly with hepatocellular injury characterized by extremely elevated aminotransferase levels, which resolved without acute liver failure or need for liver transplant referral.

## 1. Introduction

Drug-associated hepatotoxicity has been well defined in the literature. The fixed-dose Efavirenz/Emtricitabine/Tenofovir (EFV/FTC/TDF) (Atripla) is a first-line agent for the treatment of HIV but has rarely been associated with acute hepatotoxicity. We present a case of EFV/FTC/TDF-associated hepatotoxicity in a young, healthy male with HIV.

## 2. Case Report

A 24-year-old Hispanic male with a history of vertically acquired HIV presented for evaluation of right upper quadrant pain and nausea for three days. He was diagnosed with HIV at the age of four and followed routinely in the HIV clinic. His CD4 count in February 2014 (four months prior to admission) was 563 cells/mm^3^ with an undetectable viral load and similar values were found during his admission. The patient had been on a combination therapy of FTC, ritonavir, and atazanavir; however, he expressed interest in changing to a one-pill, once-a-day combination. Two months prior to admission, the patient was started on a new antiretroviral regimen of EFV/FTC/TDF. He was taking no other medications; however, he admitted to drinking at least eight shots of hard liquor at a party a few days prior to presentation. He denied any fevers, chills, sore throat, chest pain, shortness of breath, jaundice, rash, or diarrhea.

**Figure 1:**
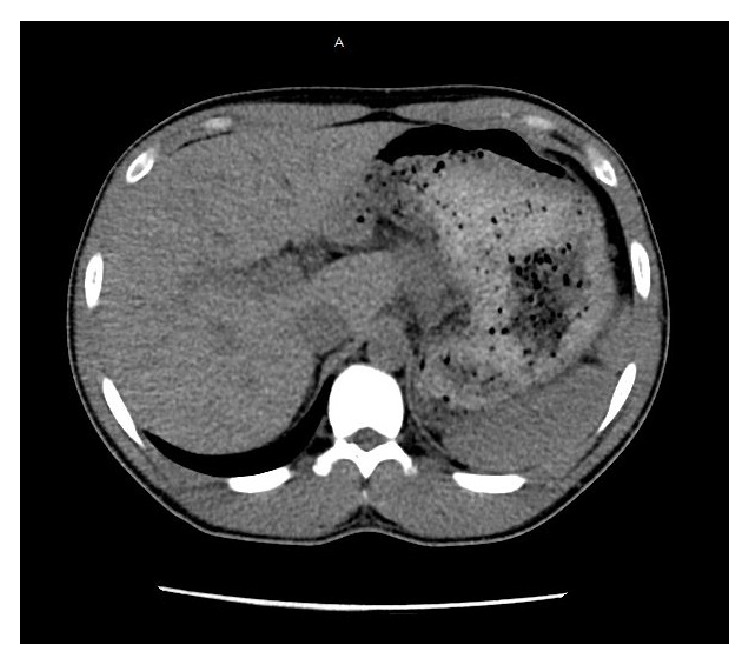
CT abdomen/pelvis without contrast showing heterogeneous liver parenchyma, no cirrhosis, and no ascites.

Upon admission, the liver enzymes were found to be elevated with an aspartate aminotransferase (AST) level of 1663 IU/L and an alanine aminotransferase (ALT) level of 1793 IU/L (Table 1). EFV/FTC/TDF was suspected to be related to the liver chemistry abnormalities and was held on the day of admission. On day two of the hospital course, he was transferred to the intensive care unit for closer monitoring because the liver enzymes continued to rise dramatically (AST 4297 IU/L and ALT 5346 IU/L). Despite the remarkably elevated aminotransferases, the total bilirubin was 0.6 mg/dL, alkaline phosphatase was 107 IU/L, and INR was 1.4, and he did not develop signs of hepatic encephalopathy. Acetaminophen level and urine drug screen were negative. Physical exam was remarkable only for mild right upper quadrant tenderness. A viral hepatitis panel was negative for hepatitis A, hepatitis B, and hepatitis C. Antimitochondrial antibodies and antinuclear antibodies were negative. Ceruloplasmin and alpha-1 antitrypsin were normal. Given the patient's normal CD4 count, undetectable viral load, and no suggestive findings on physical exam, hepatotropic viruses such as cytomegalovirus, Epstein Barr virus, herpes simplex virus, and varicella were considered less likely and therefore were not checked. Computed tomography (CT) scan showed heterogeneous liver parenchyma with no ascites and no evidence of cirrhosis (Figure 1). In consultation with poison control, the patient was started on the N-acetylcysteine protocol (10227 mg in 200 mL of D5 free water over one hour followed by 3409 mg/500 mL of D5 water over four hours followed by 6818 mg/1L D5 water over 16 hours). The patient was not treated with antibiotics during the hospital course or given any additional medications that might have slowed down the improvement of the liver biochemical tests.

The clinical condition of the patient and his liver chemistries continued to improve and obviated the need for liver biopsy or referral to a liver transplantation tertiary center. His hospital course lasted seven days and he was seen in outpatient clinic three weeks following discharge at which point repeat liver profile testing revealed an ALT of 55 IU/L and an AST of 33 IU/L. After discharge his highly active antiretroviral therapy (HAART) was changed to TDF, rilpivirine, and FTC and then to a combination of cobicistat, elvitegravir, FTC, and TDF. His liver profile remained normal several months thereafter.

## 3. Discussion

The fixed-dose combination of EFV/FTC/TDF is a first-line agent for the treatment of HIV; however few cases have reported hepatotoxicity associated with the drug [1]. Hepatotoxicity to EFV/FTC/TDF is attributed to a full-body hypersensitivity reaction and patients commonly present with rash and fever. Peripheral eosinophilia is often present. Hepatotoxicity is most commonly associated with the EFV component of Atripla [2, 3]. Although the mechanisms involved are not clear, recent evidence has pointed to a specific mitochondrial action of EFV accompanied by the induction of an endoplasmic reticulum stress/unfolded protein response in human hepatocytes [4]. Given the widespread use of EFV in the multidrug therapy for HIV infection, this mechanism of cellular response to drug-induced stress could help explain the hepatic toxicities that accompany the pharmacological treatment of some HIV patients. Endoplasmic reticulum stress triggered by EFV can be exacerbated in settings which further compromise liver function including viral hepatitis and drug or alcohol abuse [5].

Our patient presented with remarkably elevated transaminases, pointing to a pattern of hepatocellular injury rather than the more commonly associated pattern of cholestasis. He did not have a long-standing history of alcohol abuse but he had been drinking more heavily the few days prior to admission. The association of hepatotoxicity due to alcohol binging and Atripla remains unclear. This patient's liver toxicity was most likely caused by EFV/FTC/TDF and though some patients have required liver transplant, our patient recovered after four weeks of stopping the medication. In cases of severe hepatic decompensation due to suspected HAART etiology, a close collaboration of the gastroenterologist and the HIV specialist is important and recommended.

## Figures and Tables

**Table 1:** Trend of liver chemistries.

	Day 1	Day 2	Day 3	Day 5	Day 7	3 weeks after discharge
Aspartate aminotransferase (IU/L)	1663	4297	997	128	53	33
Alanine aminotransferase (IU/L)	1793	5346	2934	1315	703	55
Alkaline phosphatase (IU/L)	107	68	74	83	88	42
Total bilirubin (mg/dL)	1.4	1.3	1.4	0.6	0.3	0.3
INR	1.4	1.3	N/A	N/A	N/A	1
